# Neural Correlates for Intrinsic Motivational Deficits of Schizophrenia; Implications for Therapeutics of Cognitive Impairment

**DOI:** 10.3389/fpsyt.2018.00178

**Published:** 2018-06-05

**Authors:** Kazuyoshi Takeda, Tomiki Sumiyoshi, Madoka Matsumoto, Kou Murayama, Satoru Ikezawa, Kenji Matsumoto, Kazuyuki Nakagome

**Affiliations:** ^1^Department of Psychiatry, National Center Hospital, National Center of Neurology and Psychiatry, Tokyo, Japan; ^2^Department of Preventive Intervention for Psychiatric Disorders, National Institute of Mental Health, National Center of Neurology and Psychiatry, Tokyo, Japan; ^3^Department of Neuropsychiatry, The University of Tokyo Hospital, Tokyo, Japan; ^4^School of Psychology and Clinical Language Sciences, University of Reading, Reading, United Kingdom; ^5^Research Institute, Kochi University of Technology, Kochi, Japan; ^6^Brain Science Institute, Tamagawa University, Tokyo, Japan; ^7^National Institute of Mental Health, National Center of Neurology and Psychiatry, Tokyo, Japan

**Keywords:** intrinsic motivation, cognitive remediation therapy, schizophrenia, lateral prefrontal cortex, striatum, self-determination theory, neuroimaging, social functioning

## Abstract

The ultimate goal of the treatment of schizophrenia is recovery, a notion related to improvement of cognitive and social functioning. Cognitive remediation therapies (CRT), one of the most effective cognition enhancing methods, have been shown to moderately improve social functioning. For this purpose, intrinsic motivation, related to internal values such as interest and enjoyment, has been shown to play a key role. Although the impairment of intrinsic motivation is one of the characteristics of schizophrenia, its neural mechanisms remain unclear. This is related to the lack of feasible measures of intrinsic motivation, and its response to treatment. According to the self-determination theory (SDT), not only intrinsic motivation, but extrinsic motivation has been reported to enhance learning and memory in healthy subjects to some extent. This finding suggests the contribution of different types of motivation to potentiate the ability of the CRT to treat cognitive impairment of schizophrenia. In this paper, we provide a review of psychological characteristics, assessment methods, and neural correlates of intrinsic motivation in healthy subjects and patients with schizophrenia. Particularly, we focus on neuroimaging studies of intrinsic motivation, including our own. These considerations are relevant to enhancement of functional outcomes of schizophrenia.

## Introduction

The ultimate goal of the treatment of schizophrenia (SCZ) is recovery, a notion related to improvement of social functioning, such as employment, independent living, and interpersonal relations (1). Patients with SCZ generally show impairments of cognitive functions, e.g., verbal memory, verbal fluency, motor function, attention, working memory, and executive function. Importantly, cognitive impairments have been reported to deteriorate social functioning (1–3).

Although antipsychotic medications exert limited effects on cognitive functions (4, 5), cognitive remediation therapies (CRTs) (6, 7) and neuromodulation, such as repetitive transcranial magnetic stimulation (8) and transcranial direct current stimulation (9), have been reported to moderately improve them. CRTs represent a psychosocial intervention that aim to directly improve cognitive functions by inducing neuroplasticity (6, 7). To attain certain improvement in social functioning, it is recommended to include CRT in a comprehensive rehabilitation program along with other psychosocial treatments, such as social skills training and cognitive behavioral therapy. Recent evidence shows the importance of integrating intermediate factors, such as social cognitive function and motivation, with CRT to effectively promote social functioning (10). Motivation is generally subdivided into intrinsic and extrinsic ones. Intrinsic motivation is subject to internal values, such as interest and enjoyment (10, 11), whereas extrinsic motivation is generated by external factors, such as reward and punishment. Intrinsic, rather than extrinsic motivation is considered to play a key role in enhancing the effect of psychiatric rehabilitation.

Among several types of CRT, the Neuropsychological Educational Approach to Cognitive Remediation (NEAR), developed by Medalia and Choi (12), focuses on enhancing intrinsic motivation. The program includes a group session, which bridges the cognitive training with daily life (12, 13). To evaluate intrinsic motivation for the intervention, self-report measures of intrinsic motivation, such as the Intrinsic Motivation Inventory (IMI), have been developed based on the self-determination theory (SDT) (14). Previous studies indicate that the IMI modified for SCZ patients (IMI-SR) (15) reflects internal value of a specific task or activity (16–18). Monitoring intrinsic motivation during the CRT is critical to determine whether the intervention is effective for each patient and/or whether a modification is necessary. For example, Silverstein (19) reported that the increase in intrinsic motivation was positively correlated with improvement in social functioning. However, examinations of intrinsic motivation using a self-report scale, such as IMI, may not be accurate, because participants' answers may be influenced by response bias caused by social desirability and self-monitoring capacity. In fact, cognitive deficits of SCZ have been associated with decreased activity in the lateral prefrontal cortex (LPFC) (20, 21), which may lead to a poor self-monitoring capacity. Therefore, it is essential to develop objective scales to monitor intrinsic motivation, which are feasible in clinical settings. For this purpose, understanding the neural basis of intrinsic motivation is necessary.

In healthy subjects, not only intrinsic motivation, but also autonomous types of extrinsic motivation are important for enhancing learning and memory (22). This is based on the SDT, suggesting that motivation comprises of several steps in a continuum of relative autonomy. Most autonomous types of motivation are intrinsic in nature, while extrinsic motivation varies from autonomous to controlled ones. In the psychosocial therapy of patients with SCZ, enhancing autonomous types of extrinsic motivation may lead to greater improvement of cognitive and social functioning.

In this paper, we reviewed the literature on the following topics: (1) the definition of intrinsic motivation based on the SDT, (2) the assessment of intrinsic motivation, (3) the role for intrinsic motivation in the therapeutics of cognitive impairment and (4) the neural basis of intrinsic motivation. Specifically, we summarize some findings on the neural correlates of intrinsic motivation estimated by neuroimaging in healthy subjects and patients with SCZ.

## Definition of intrinsic motivation based on self-determination theory

Ryan and Deci (22) defined intrinsic motivation as “the inherent tendency to seek out novelty and challenge, to explore and investigate, and to stretch and extend one's capacities.” On the other hand, extrinsic motivation is affected by external control, such as acquisition of reward or avoidance of a punishment. Among several theories to explain intrinsic motivation, such as the empirical drive theory, psychodynamic drive theory, and effectance motivation (23), the SDT (22, 24) is considered comprehensive for understanding intrinsic motivation. The SDT, based on organismic and humanistic principles, proposes a multidimensional theory of motivation. It has been developed out of the idea that intrinsic and extrinsic reasons for behaving lead to differential levels of performance and well-being for individuals (14, 24). Specifically, intrinsic motivation is suggested to be more closely associated with better performance, persistence, and well-being, and more accurately predict the adjustment of behavior, compared to extrinsic motivation (22, 24).

Previous studies have reported that intrinsic motivation varies under different conditions (14). Events such as the provision of positive feedback (25–27) and choice (28) enhance intrinsic motivation, whereas negative feedback (29, 30), deadlines (31), extrinsic motivation (32, 33) and other external impositions (34) generally diminish intrinsic motivation. Particularly, the undermining effect is known to exist between intrinsic and extrinsic motivation. The SDT indicates it is essential to satisfy three basic psychological needs: autonomy, competence, and relatedness for enhancement of intrinsic motivation (22). The need for autonomy is the sense that one's behavior should be self-determined, and includes the desire to take responsibility for one's own actions. On the other hand, the need for competence means the desire to feel the confidence that “I could do it if I tried,” and to prove oneself to others. Finally, the need for relatedness refers to the desire to build and maintain a good relationship with other people in the community, and to attain a sense of solidarity (22).

Although, motivation is traditionally subdivided into intrinsic and extrinsic motivation, it has been suggested that motivation is not fully explained by this dichotomy (22). For example, when a student studies a foreign language because it is a required course, his action is extrinsically motivated based on external control. When a student studies a foreign language for preparation to study abroad, it is also extrinsically motivated because the action is desirable as a means of achieving goal, not solely for interest or enjoyment. These two examples are considered to illustrate different types of extrinsic motivation in terms of the degree of autonomy. In this way, extrinsic motivation can dynamically shift according to the degree of internalization, or the process of taking in a value.

**Figure 1:**
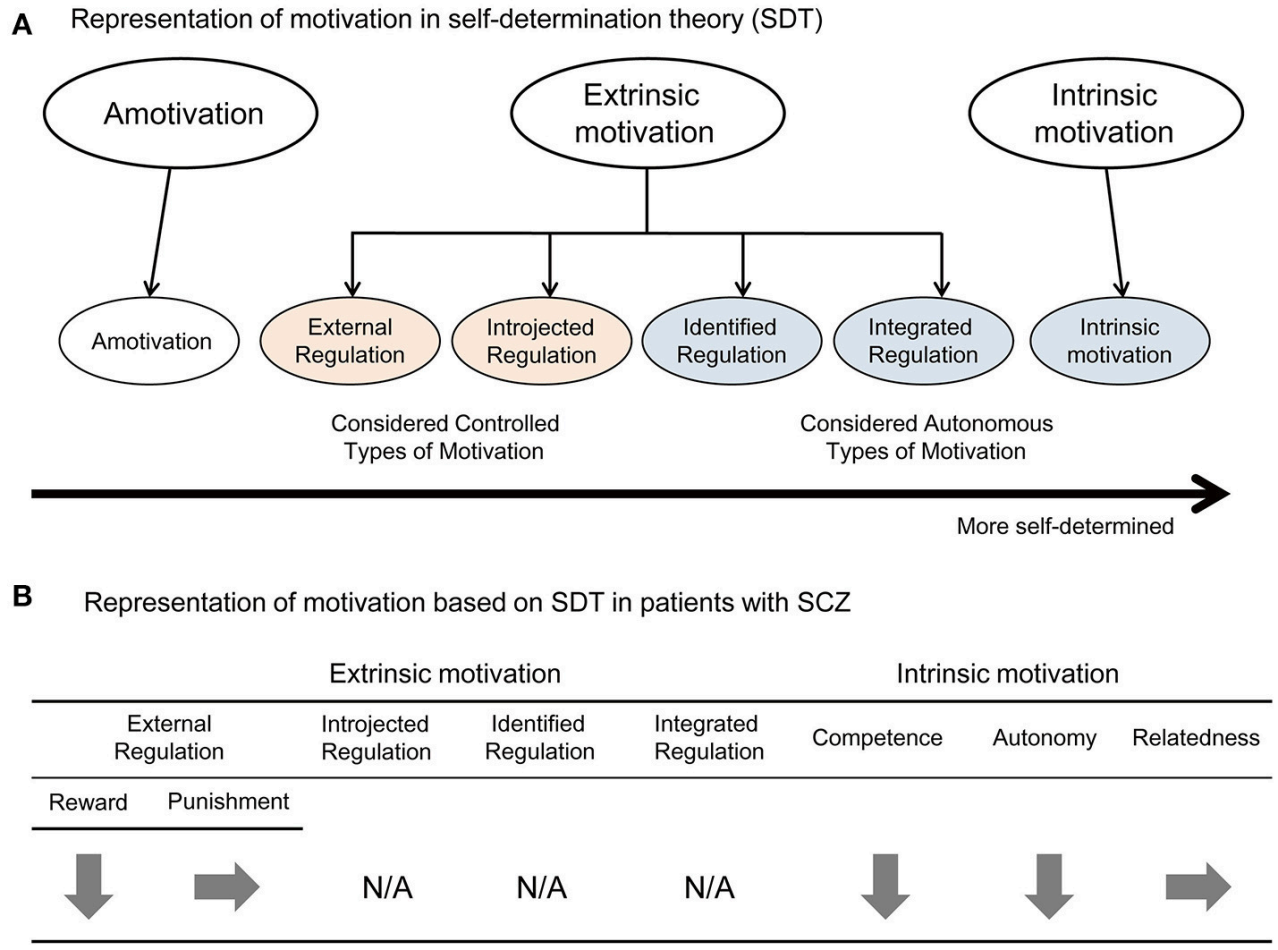
**(A)** Representation of motivation in self-determination theory (SDT). This figure modified from Ryan and Deci (22). **(B)** Representation of motivation based on SDT in patients with SCZ. This shows data from Gard et al. (38) applied to the continuum in SDT.

Based on this background, the SDT proposes a dimensional representation of motivation that is comprised of several steps along a continuum of relative autonomy (22). Figure 1A illustrates six types of motivation in the continuum of SDT. Among them, intrinsic motivation is the most self-determined and autonomous motivation. Extrinsic motivation is subdivided into four types, according to the degree of autonomy. Of the four types, external regulation is considered the most controlled type (22) in which a behavior is motivated by external control (acquisition of a reward or avoidance of a punishment). For example, when a student is rated by the academic self-regulation questionnaire (SRQ-A) (35), “Why do I do my homework?”, the answer “Because I'll get in trouble if I don't” exemplifies external regulation. Introjected regulation is also considered a controlled type whereby a person's behavior is motivated by goals such as avoiding shame or anxiety or to maintain pride (22). For example, a student would answer “Because I want the teacher to think I'm a good student” in the same question. On the other hand, the stage of “identified regulation” categorized as more autonomous, whereby an action is motivated because of its value toward a goal, not because it is enjoyable or interesting (22). For example, students answer “Because it's important for me to do my homework” in the same question. Finally, integrated regulation is considered to be the most autonomous among the extrinsic motivation subtypes. In this type, a person's action is motivated because it is desirable and natural to do it, not necessarily because it is enjoyable or interesting (22). For example, students would answer “Because I want to do my homework” in the same question. Identified regulation and integrated regulation are known to improve performance, decrease dropout, and enhance learning, relative to more controlled types of regulation (36). Moreover, as Deci and Ryan (22) noted, the autonomous types of extrinsic motivation have been associated with the greater task engagement, better performance, and more learning.

Although studies of SCZ have focused on the impairment of intrinsic motivation (16, 37), little is known about continuous motivation deficits based on the SDT. In this respect, Gard et al. (38) examined whether each type of motivation in patients with SCZ was different from that in healthy subjects using the Ecological Momentary Assessment, a semi-structured interview. Goal-driving motivation in each individual was rated on a scale of specific anchors (0–3), based on the participant's response to “What goal do you have in the coming few hours?” and “What is the most important reason for having this goal?” (38). They assessed intrinsic motivation on the basis of three psychological needs, and also analyzed extrinsic motivation by separating the requirements of a reward and avoidance of a punishment. They found that the need for relatedness and extrinsic motivation based on punishment was not different between SCZ patients and healthy subjects, although the former showed less need for autonomy and competency, and extrinsic motivation based on reward (38). Figure 1B summarize the continuum in the SDT. Thus, people with SCZ may want to attain a good relationship with other people, which is hindered by impairment in social cognition, leading to the difficulty to link extrinsic reward to their actions (39, 40). This suggests that SCZ patients, perhaps due to repeated negative experiences, tend not to require extrinsic rewards and are sensitive to punishment (41).

In summary, it is important to develop treatment that enhances autonomy, competence, and sensitivity to extrinsic reward (38). Future studies will be required to understand which level of extrinsic motivation is impaired in SCZ, and how this relates to social functioning.

## Behavioral assessments of intrinsic motivation in schizophrenia

**Figure 2:**
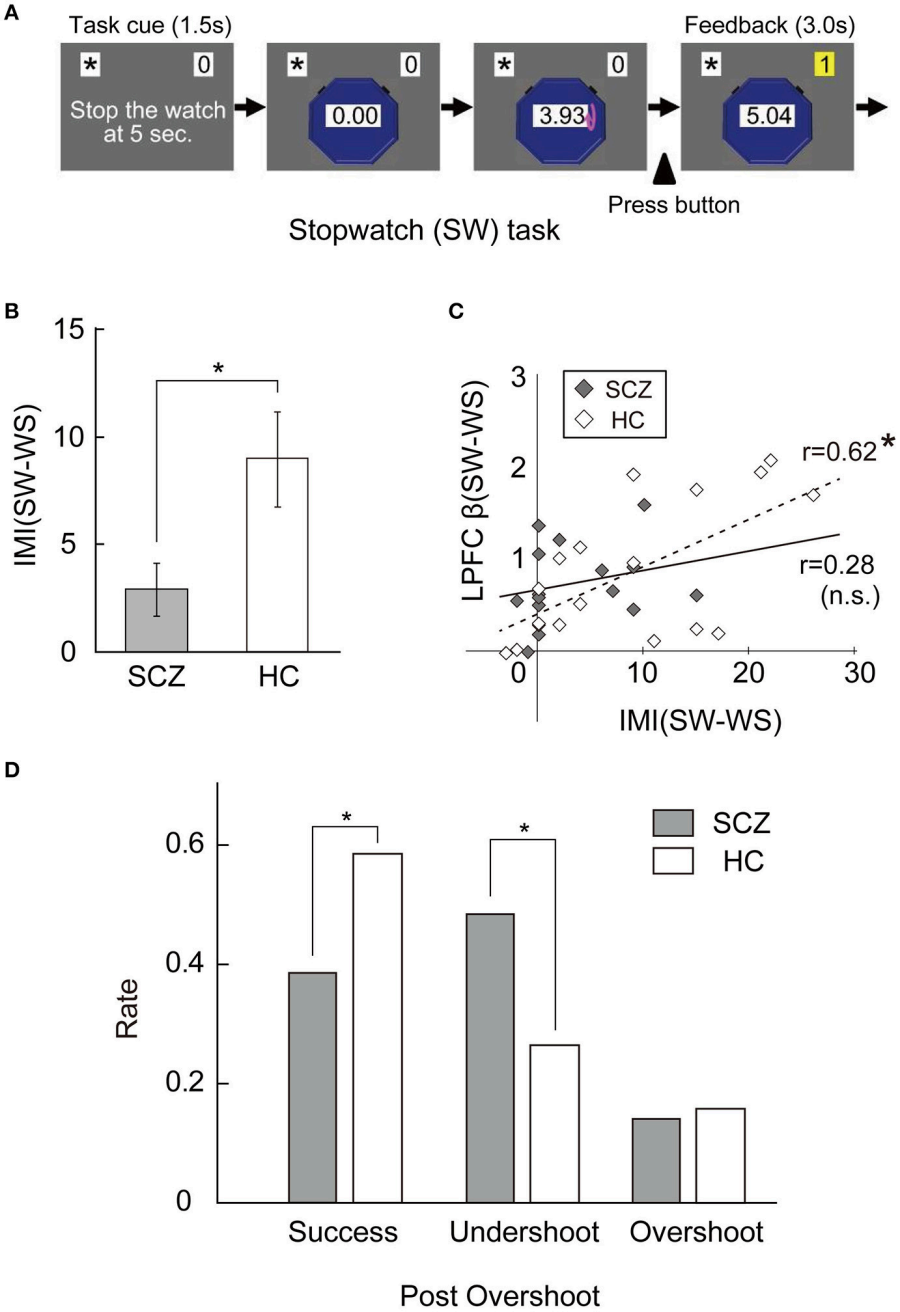
**(A)** The stopwatch (SW) task. **(B)** The differences in IMI-SR between SW and WS tasks showed a significant between-group difference. (Mann–Whitney *U*-test, error bar: SEM, ^*^p < 0.05). **(C)** In HC, the neural activity in LPFC was positively and significantly correlated with the index of intrinsic motivation, whereas not in SCZ. (Pearson, ^*^p < 0.0125, n.s.: not significant). **(D)** Comparison of the performance level following the Overshoot between SCZ and HC. There was a significant main effect of Post-Overshoot (Success vs. Undershoot vs. Overshoot) (*p* < 0.001), and a significant interaction between Post-Overshoot and Group (SCZ vs. HC) (*p* = 0.01). The secondary analysis for each Post-Overshoot trial revealed a significant main effect of group for Success (*p* = 0.01) and Undershoot (*p* = 0.01). These figures modified from Takeda et al. (54).

The free-choice paradigm is known as a representative measurement of intrinsic motivation. In this paradigm, people can freely try different tasks, including a target task for a brief period when they believe nobody is observing their behavior. The number of trials during the free-choice period is used as an index of intrinsic motivation (32, 33). For example, Murayama et al. (42) developed the stopwatch (SW) task (Figure 2A). In this task, a SW appears on the monitor, and starts automatically; subjects are required to stop the SW within 50 ms of the 5-s time point by pressing a button (42). As a control task, they also used the watch-stop (WS) task, in which subjects press a button after the SW automatically stopped. A computer and a few booklets were set on the table in the room and participants could freely spend three minutes in this room (42). They could either play the SW or WS task, or read booklets as they like. Although subjects believed that nobody observed their behavior during this period, the number of trials was recorded by the computer program (42). By comparing the number of trials between the SW and WS tasks, the intrinsic motivation level was evaluated. The IMI is a self-report measurement of intrinsic motivation derived from the SDT (14). The IMI (27) comprises of 6 subscales (interest/enjoyment, effort, value/usefulness, pressure/tension, relatedness, and choice) and 54 items, and is effective for evaluating the level of intrinsic motivation for various activities, such as sports, school, medical procedures, and laboratory tasks (43–46).

Both measures described above have limitations (47). The results obtained from the free-choice paradigm may not always reflect intrinsic motivation because it is difficult to distinguish this from other processes such as persistence and preparing for future trials (47). Likewise, the self-report measures may not accurately capture the dynamic aspect of intrinsic motivation. Since participants are usually unable to report the intrinsic motivation during performance on a task, they are likely to report it afterwards on the basis of their memory (47). Based on these limitations, relatively few studies have measured both of them.

As discussed, little has been reported on the assessment of intrinsic motivation using the free-choice paradigm. On the other hand, Tobe et al. (48) used the General Causality Orientations Scale (GCOS) to examine the property of motivation in SCZ patients. This scale evaluates the degree of three types of orientation (autonomy, control, impersonal orientations) based on the SDT. Although scores on the control and impersonal orientations were not different between SCZ and healthy control groups, those on autonomy was significantly lower for the SCZ group compared to the control group. These findings suggest that the GCOS provides a valid measure to evaluate a declined of intrinsic motivation in SCZ patients. On the other hand, Choi et al. (15) modified the original IMI to assess the interventional effect of various psychosocial therapies in patients with SCZ, and named it IMI-SR. The IMI-SR consists of three subscales (interest and enjoyment, values, and choice) and a total of 21 questions (15). Previous studies indicate that the scale is effective to evaluate intrinsic motivation not only in association with the CRT, but also other trainings (15–18). However, participants' answers may be influenced by response bias caused by self-monitoring capacity.

## Role of intrinsic motivation in the therapeutics of cognitive impairment in schizophrenia

In psychosocial therapies such as CRT, the balance of intrinsic motivation and extrinsic motivation to maximize the therapeutic effects is the critical issue. In SCZ, it has been shown that reward-based learning is ineffective because patients are less sensitive to positive feedback than are healthy controls (20, 49–51). On the other hand, when patients are intrinsically motivated to engage in a treatment program, they actively participate because they feel the activity itself is interesting and enjoyable (15). Therefore, it is critical to heighten the level of autonomy and self-efficacy, which support intrinsic motivation. Accordingly, Nakagami et al. (52) examined whether or not intrinsic motivation affects functional outcomes. They found that intrinsic motivation directly enhanced the neurocognitive improvement after CRT, and is an essential factor to improve social functioning (52). Moreover, they (53) showed that intrinsic motivation dynamically improves through the intervention, which was closely correlated with improvement in social functioning.

It is essential to enhance intrinsic motivation during the CRT. To enhance motivation, the bridging session is implemented, in which we try to link the training session to everyday activities. For example, the patients' group would discuss what benefit does training of spatial memory brings, and finally reach a conclusion that it is useful when we need to remember the location of an item on the shelf while working as a clerk. As this process aims to improve understanding the usefulness of the training, it does provoke autonomous types of extrinsic motivation, which are identified and/or integrated. Also, Silverstein (19) reported that extrinsic motivation is related to the improvement of cognitive impairment, and both intrinsic motivation and extrinsic motivation are essential for desirable outcomes in CRT. This finding is in line with the suggestion that extrinsic motivation may be critical to enhance the initiation of behavior, and to produce intrinsic motivation for maintaining it (19). Previous studies reported that extrinsic motivation, such as reward, are used in supportive interpersonal relations to promote the needs for competence and autonomy (55, 56). These items can be an integral component of cognitive enhancement techniques based on self-identified goals (57). In fact, positive outcomes related to extrinsic motivation are observed in learning (58) and treatment response (59). In addition, neural activity in brain areas related to cognitive function has been suggested to be modulated by anticipation and appearance of reward (49). Moreover, the undermining effect is observed if people are fully and intrinsically motivated to perform a specific activity and task, and adequately expect extrinsic reward. Since SCZ patients tend to have relatively low intrinsic motivation, data from studies of the undermining effect in healthy subjects may be absent in SCZ patients (19). These considerations suggest that it may be worth incorporating extrinsic motivation such as reward into the CRT.

In summary, it will be essential to assess to what extent different types of extrinsic motivation, and intrinsic motivation are impaired in each patient for enhancing cognitive and social functioning.

## Neural basis for intrinsic motivation in schizophrenia

It is challenging to set up situations that lead participants to experience a sense of competence, i.e., manipulation of intrinsic motivation (60, 61). In recent neuroimaging studies, intrinsic motivation has been examined in various ways (Table 1).

**Table 1:** Studies in neural correlates of intrinsic motivation using fMRI.

**Study**	**Subjects**	**Sources of intrinsic motivation**	**Task**	**Paradigm and data detection**	**Related brain area**
^*^(42)	HC: 28	Interest and enjoyment	Game like task (i.e., stopwatch task)	In the stopwatch (SW) task, subjects are required to stop the SW by pressing a button by myself. In the control task, subjects only need to press a button after a SW automatically stopped. The SW task is more interesting than the control task. They compared the activity during task cue period that indicates which of the two tasks will be displayed.	Striatum LPFC
(62)	HC: 35	Self-determination	Game like task (i.e., stopwatch task)	In self-determined-choice condition, participants performed the SW task by selecting freely one of two SWs with different appearances. In forced-choice condition, participants performed the SW task using an automatically designated SW. They compared the activity during the feedback period after the button press between success and failure in each condition.	MPFC
(63)	HC:19	Curiosity	Game like task (i.e., trivia questions)	Participants was presented the trivia questions. After they read them, they reported the level of curiosity of them. Authors examined the activity during the presentation period of the trivia questions by the difference of the level of curiosity.	Striatum LPFC
(64)	HC:24	Curiosity	Game like task (i.e., trivia questions)	After the trivia question was presented, participants anticipated the presentation of the answer. They investigated the activity during the anticipation period by the difference of the level of curiosity	Substantia nigra/ventral tegmental area
(67)	HC:10	Interest and enjoyment	Game like task (i.e. reading situation regarding IM and EM)	One of three phases (IM, EM, and Neutral) was selected. Participants read the selected phase of situation and replied by pressing a button whether they want to do it. Authors compared the activity during the presentation of the situation between IM and EM phase.	AIC
(61)	HC:16	Interest and enjoyment	Game like task (i.e. reading situation regarding IM and EM)	One of three phases (IM, EM, and Neutral) was selected. Participants read the selected phase of situation and reported how much they want to engage in it. Authors compared the activity during the presentation of the situation between IM and EM phase.	AIC
(68)	HC:22	Curiosity	Game like task (i.e., curiosity-inducing questions)	Participants were presented randomly selected question, and was asked to think of the correct answer, and reported how interesting the question or anagram was. Authors compared the activity during the presentation of the question between curiosity-inducing question and non-curiosity-inducing question.	Striatum AIC
(54)	SCZ:18 HC:17	Interest and enjoyment	Game like task (i.e., stopwatch task)	In the stopwatch (SW) task, subjects are required to stop the SW by pressing a button by myself. In the control task, subjects only need to press a button after a SW automatically stopped. The SW task is more interesting than the control task. We examined the activity during task cue period that indicates which of the two tasks will be displayed.	Striatum LPFC

*HC, Healthy control; SCZ, schizophrenia; IM, intrinsic motivation; EM, extrinsic motivation; LPFC, Lateral prefrontal cortex; MPFC, Medial prefrontal cortex; AIC, Anterior insular cortex*.

* *Although Murayama et al. (42) examined the neural basis of the undermining effect, they also found the brain area related to intrinsic motivation in control group*.

Some studies have compared neural activity in intrinsically enjoyable game-like tasks and less enjoyable tasks. For example, Murayama et al. (42) observed that participants played the SW task more frequently than the WS task during the free-choice period, and found greater neural activity in the anterior striatum and LPFC during the SW task than the WS task. This finding suggests that these two areas constitute a neural system related to intrinsic motivation. Moreover, Murayama et al. (62) examined the neural mechanism related to self-determination by comparing neural activity during the SW task in two conditions. One was a self-determined-choice condition in which participants were freely required to select one of two SWs with different appearances, and the other was a forced-choice condition in which they were required to perform the SW task using an automatically designated SW (62). Whereas the activity of ventromedial prefrontal cortex (VMPFC) was markedly reduced in response to failure feedback compared to success feedback in the forced-choice trials, the activity levels were similarly high in the responses to the feedback between success and failure in the self-determined-choice trials. This suggests the neural activity in VMPFC is closely related to information processing regarding self-determination (62).

Kang et al. (63) investigated the neural mechanism related to curiosity by examining the relationship between neural activity when participants processed the trivia questions and the degree of curiosity about them. They found that activities in the caudate nucleus and LPFC were increased when participants experienced a higher level of curiosity, and that the increase of the activity in these brain areas was associated with improvement of memory (63). They pointed out that the caudate nucleus and LPFC are involved in memory encoding, and that intrinsic motivation is associated with enhanced learning (63). To assess more directly the relation between curiosity and learning, Gruber et al. (64) examined whether memory for task-relevant or for task-irrelevant information was improved depending on the level of curiosity by using trivia questions similar to those used by Kang (63). In this study, face stimuli were used as task-irrelevant information. When the level of curiosity was high, the recalls of both task-relevant and task-irrelevant information were improved. Moreover, they found that increased activation in the substantia nigra/ventral tegmental area (SN/VTA) and the hippocampus was related to the enhancement of memory (64). These findings indicate that the neural activity related to motivation that includes the striatum and LPFC (64, 65) may enhance the learning potential (60, 62–64, 66).

To determine whether the neural system of intrinsic motivation is different from that of extrinsic motivation, Lee's group (61, 67) proposed unique neural mechanisms related to intrinsic motivation. The authors compared neural activities when subjects imagined an action based on intrinsic motivation vs. those when they imagined the same action based on extrinsic motivation. They found that the anterior insular cortex (AIC) was activated to a greater extent in the intrinsic motivation situation compared to that of extrinsic motivation, suggesting that the AIC plays a role in intrinsic motivation (61, 67). Moreover, Lee and Reeve (68) examined neural activities when subjects actually performed the action based on intrinsic motivation in different levels of autonomy. They found that not only AIC but also the striatum was activated and that the functional connectivity between these brain areas was enhanced, suggesting that both brain regions are important for generating intrinsic motivation (68). The insular cortex plays a major role in processing emotion and feeling regardless of valence and integrating emotionally salient information and forming subjective emotional feelings (69, 70). The activity of the insular cortex was increased in performing self-generated behaviors compared with other behaviors (71, 72). In addition, its activity is related not only to who initiates and regulates certain behavior but also whether the behavior is generated from the “pure self” rather than from social influence (61, 67, 68).

In an electroencephalogram (EEG) study, Meng and Ma (73) examined the effect of autonomy using two time-estimation tasks with equal difficulty. In the choice condition, participants freely selected a time-estimation task, which requires subjects to indicate the end of the prespecified interval by pressing a button. In the no-choice condition, they performed the task automatically selected by a computer. They found a larger feedback-related negativity (FRN) in the choice condition compared to the no-choice condition. Moreover, Jin et al. (74) investigated the neural basis of intrinsic motivation by examining the neural disparity between the SW and WS tasks using event-related potentials. In the task cue period, the N2 amplitude in the SW task was smaller than that in the WS task. In the outcome period, smaller FRN amplitudes and lager P300 amplitudes were observed in the SW task compared to those in the WS task. Although the findings about the FRN in the two studies somewhat contradict with each other, they suggest that intrinsic motivation is measurable by means of event-related potentials, such as N2, FRN, and P300.

To clarify the neural mechanism of intrinsic motivation impairment in people with SCZ, we studied the neural activity in the striatum and LPFC of SCZ patients while they performed the SW task (Figure 2A) (54). Specifically, we compared the brain activity measured by fMRI and behavioral data between SCZ patients and healthy control (HC) participants (54). Firstly, scores of IMI-SR in the two tasks showed a significant between-group difference, so that people with SCZ were less intrinsically motivated for the SW task (Figure 2B) (54). Similarly, cue-related activity in the striatum was lower in SCZ compared to HC. Secondly, a positive relationship was noted between the cue-period activity in LPFC and the level of intrinsic motivation in HC subjects was absent in SCZ patients (Figure 2C) (54). Thirdly, although the performance level per se was not significantly different between the two groups, the capacity of correction after error trials was somewhat different. To analyze it, we first distinguished the error trials into two types; “Undershoot” stands for the error when the button press is too fast (< 4.95 s) while “Overshoot” stands for the error when the button press is too slow (>5.05 s). For example, in the trial after Overshoot, participants were required to regulate button press speed so as not to press the button too soon for success. As a result, the Success and Undershoot rates following Overshoot showed a significant between-group difference (Figure 2D), whereas those following Undershoot were not different. These results suggest that the regulation of button press after Overshoot, which is considered as a form of cognitive control, is impaired in SCZ patients (54). In addition, a positive relationship between the cue-period activity in LPFC and the Success rate after Overshoot was observed in healthy control subjects, but not in SCZ patients (54).

The lack of relationship between intrinsic motivation and LPFC activity suggests that SCZ patients do not adequately regulate actions because of impaired prefrontal activity (54). This is also supported by the absence of associations between the capacity to regulate response and LPFC activities. These observations are consistent with previous findings about reward processing. Despite the argument that SCZ patients exhibit a hedonic response comparable to that of HC subjects (75–77), these patients elicit lower motivation to initiate and retain an action (40, 78). Our finding suggests a failure to mediate between prediction of reward and action in people with SCZ (79, 80). In addition, it is suggested that information processing related to reward expectation is processed in the striatum and PFC (81), and the LPFC is a central brain area for enhancing actions related to reward expectations (82, 83). The PFC and the striatum are closely linked via the frontostriatal loops (84–87). Previous studies indicated that the neural activity in the ventral striatum to the reward expectation was impaired in SCZ patients (88–90). Based on these findings, it appears that SCZ patients have difficulty generating adequate action control in response to reward because of LPFC impairment.

Taken together, SCZ patients show motivation disturbances due to a weakened link between the reward expectation and goal-directed behavior which is associated with altered functions of LPFC and the striatum. Of particular importance is the difficulty of SCZ patients to adequately regulate actions on the basis of intrinsic motivation because of impaired prefrontal activity. Whether the neural system related to intrinsic motivation is different from that of extrinsic motivation remains unclear, which requires further research. For the greater chance of the success for CRT treatments, it will be beneficial to further explore the biological basis of intrinsic motivation and develop the methods to detect its individual differences.

## Future directions

Based on neuroimaging data from our studies, we are interested in discovering the ways to restore reduced neural activities in the striatum and LPFC. This strategy may lead to enhancement of intrinsic motivation and improvement of social functioning in people receiving a psychosocial treatment such as CRT. On the other hand, it has been reported that neural activity in LPFC and performance on cognitive tasks with high demands are impaired in SCZ patients (20, 21). These findings suggest that decreased activity of LPFC contributes to the impairment of both cognition and intrinsic motivation, leading to the difficulty in effectively improving cognitive impairment and social functioning. In electroencephalogram studies, enhanced frontal gamma-band oscillations have been associated with better performance in healthy subjects, while both reduced and excessive gamma-band oscillations have been suggested in patients with SCZ (91–93). Interestingly, rTMS has been reported to reduce abnormal gamma oscillations in patients with SCZ, whereas it increases gamma activity in healthy subjects during a cognitive task, which may be related to homeostatic plasticity (94). As this finding suggests that impaired prefrontal function can be modified by neuromodulation, the combination of CRT and neuromodulation may be one of the options to enhance social functioning.

At present, we have no objective and feasible scales of intrinsic motivation. However, by focusing on neural activity in LPFC evaluated in our recent studies (54) we may be able to develop a biological tool that can be applied in the clinical field. Near-infrared spectroscopy (NIRS) can be one of such techniques. It is non-invasive and can be measured under a restraint-free environment, thus suitable for psychiatric patients. The validity of NIRS has been indicated by significant correlations between fMRI BOLD signals and NIRS oxygenated hemoglobin (oxy-Hb) concentrations in the frontal area (95, 96). Using multi-channel NIRS, Pu et al. (97) reported that an increase in oxy-Hb concentrations in DLPFC is positively correlated with the interest and motivation scores in the Social Adaptation Self-Evaluation Scale in healthy subjects. This finding suggests that the NIRS signal in LPFC may provide an objective scale of intrinsic motivation in SCZ.

Although intrinsic motivation is considerably important to alleviate cognitive impairment, it is not easy to enhance intrinsic motivation of SCZ patients. Some studies report that extrinsic motivation induced by monetary rewards is useful to enhance the effect of CRT. According to the SDT, autonomous types of extrinsic motivation may switch to intrinsic motivation through the practice of CRT. The assessment of the construct of motivation in terms of levels of autonomy may provide useful information to achieve the maximum effect of CRT on cognition in patients with schizophrenia.

## Conclusions

We reviewed putative neural correlates of intrinsic motivation revealed by neuroimaging data. In spite of previous attempts, we have not yet established objective tools to monitor the degree of intrinsic motivation in each patient, which requires further investigations. Moreover, the development of CRT incorporating enhancement of intrinsic motivation, as well as autonomous types of extrinsic motivation, may be important, depending on the tendency for intrinsic motivation. These efforts are likely to enhance cognitive and social functioning in patients with SCZ.

## Author contributions

KT drafted the manuscript. All authors critically reviewed the manuscript and approved the final manuscript.

### Conflict of interest statement

The authors declare that the research was conducted in the absence of any commercial or financial relationships that could be construed as a potential conflict of interest.
